# Blood sample tube transporting system versus point of care technology in an emergency department; effect on time from collection to reporting? A randomised trial

**DOI:** 10.1186/1757-7241-20-71

**Published:** 2012-10-08

**Authors:** Birgitte Nørgaard, Christian Backer Mogensen

**Affiliations:** 1 Emergency Department, Kolding Hospital, a part of Lillebaelt Hospital, Skovvangen 2-8, Kolding, 6000, Denmark

**Keywords:** POCT, Tube transporting system, Emergency department, Randomised trial

## Abstract

**Background:**

Time is a crucial factor in an emergency department and the effectiveness of diagnosing depends on, among other things, the accessibility of rapid reported laboratory test results; i.e.: a short turnaround time (TAT). Former studies have shown a reduced time to action when point of care technologies (POCT) are used in emergency departments. This study assesses the hypothesis, that using Point of Care Technology in analysing blood samples versus tube transporting blood samples for laboratory analyses results in shorter time from the blood sample is collected to the result is reported in an emergency department.

**Methods:**

The study was designed as a randomised controlled trial with consecutive allocation into two groups and rated 1:1. Blood samples were collected on all included patients and then randomised into either POCT analyses or tube transporting for central laboratory analyses.

**Results:**

Blood samples from a total of 319 patients were included.

The mean time from collecting to reporting was 24 minutes for the POCT analysis and 70 minutes for the tube transported analysis. An unpaired Students t-test showed a significant reduction in time from collecting to reporting using POCT (p<.0001).

**Conclusion:**

We found a significantly reduced time from collecting to reporting using Point of Care Technology (POCT) in an emergency department compared to tube transported blood samples for central laboratory analyses.

## Background

Time is a crucial factor in an emergency department and the effectiveness of diagnosing depends on, among other things, the accessibility of rapid reported laboratory test results; i.e.: a short turnaround time (TAT). Reviewing the literature, the definition of turnaround time is ambiguous, but Lundberg has described the “total testing circle” with the following steps: ordering, collection, identification, (at several stages), transportation, separation (for preparation), analysis, reporting, interpretation and action. Above all, it is mandatory to reduce the time to action in order both to provide fast and focused treatment for the patients and to ensure a continuous flow in the emergency department and thus avoid crowding and bottleneck problems. Former studies have shown a reduced time to action when point of care technologies (POCT) are used in emergency departments [1,2] and new POCT systems have been developed with an analysis quality comparable to the central laboratory quality [3]. However, recent development in tube systems especially designed for direct transport of blood test tubes has resulted in new rapid and cost effective systems which do not affect the results of analysis [4].

We therefore decided to test the time used from collection of blood samples to the reporting of analysis results in the two different methods of handling blood samples: the local use of high quality point of care technology versus newly developed tube transporting system and central laboratory analysis. This study assesses the hypothesis, that using point of care technology reduces the time compared to tube transporting blood samples and central laboratory analyses in an emergency department.

## Methods

The study was carried out as a randomised controlled trial at the Emergency Department (ED), Kolding Hospital in Denmark, during a four months period in 2010. All patients admitted to the emergency department during the project period and with a request for C-reactive protein (CRP) analysis were included, regardless of age or gender. Patients suspected for appendicitis or meningitis were excluded since these patient groups followed special fast track procedures. Blood samples were collected when the medical laboratory technician responsible for the project was on day shift, with a break during the summer shutdown period.

**Figure 1:**
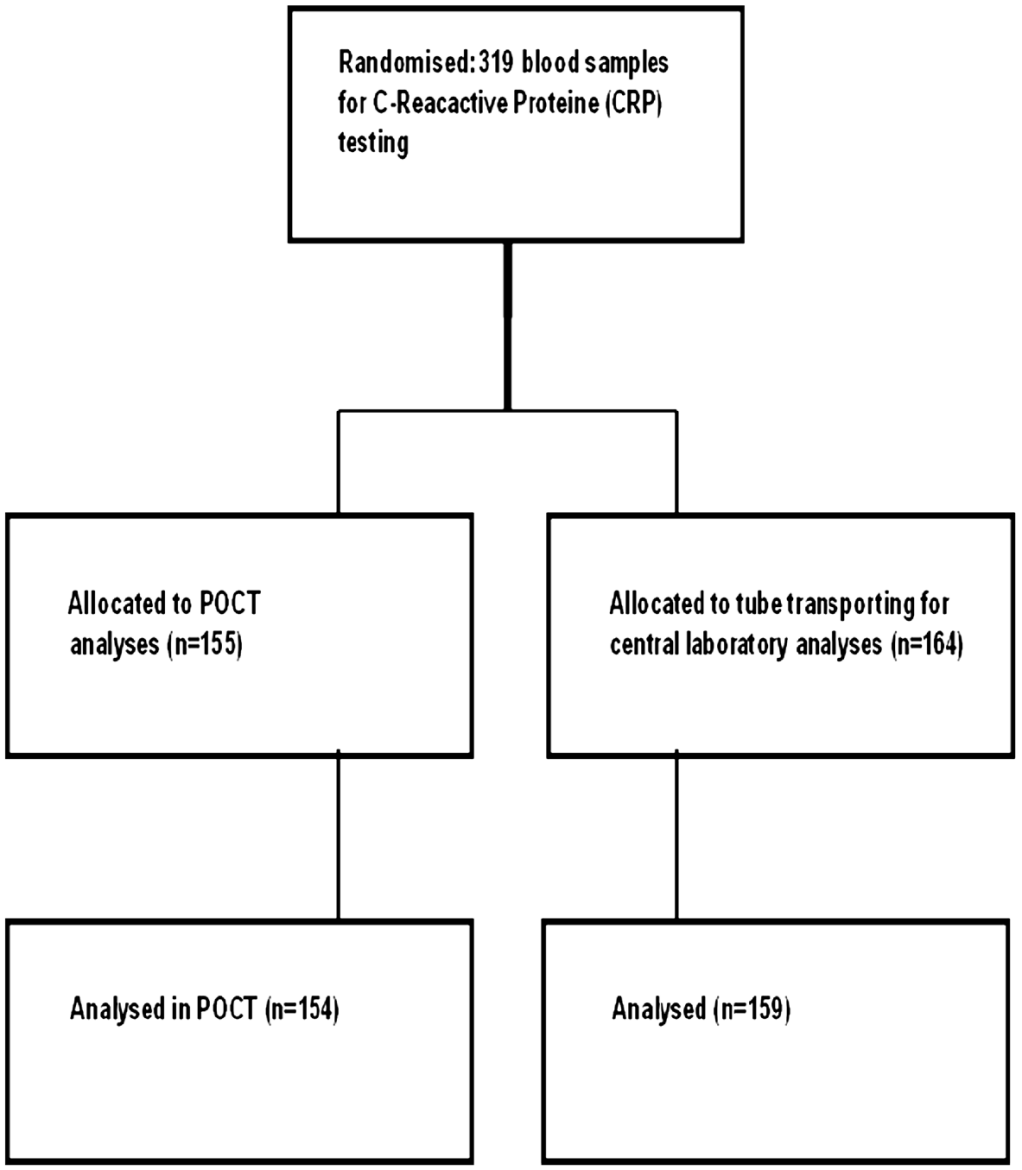
Flow chart of the allocation process stating the exact numbers in the two groups.

Blood samples for CRP analyses were collected from all included patients and then randomised into two groups: Group 1 to be analysed near-patient in the department by point of care technology (the POCT group) and Group 2 to be sent by tube transporting for conventional laboratory analysis (the TTCL group). Randomisation was performed by generation of numbers divided in 20 groups of digits 1 or 2 (Open Epi random program, http://www.openepi.com). The allocation process and distribution is shown in Figure 1.

Trained laboratory technicians were responsible for both inclusion and registration based on the pre-designed randomisation protocol, and also for the POCT-analyses and tube transporting, besides the conventional steps in the total testing circle, as mentioned above.

The POCT analyses were performed in an AQT-90 (Radiometer). The AQT-90 was developed for high quality laboratory tests using a time-resolved fluorescence method [5] with an estimated analytic time of 18 minutes. The test results were automatically transferred into the laboratory documentation system part of the medical records with a few seconds of delay. The tube transporting system was a Tempus600® (Timedico), which has recently been developed especially designed for safe and efficient blood sample transporting with a speed of 7m/s [6]. The transport distance from the ED to the central laboratory was approximately 200 m. At the time of dispatch of the blood sample the central laboratory staff was alerted by an audio signal. On arrival at the central laboratory the blood test was manually transferred to the analysing device, a Modular P800 (Roche Diagnostics) and reported automatically in the same way in the medical records as the POCT analysis. The Modular P800 has an estimated analytic time of 20 minutes for CRP (including centrifuging).The primary outcome was the time used from the blood sample was collected from the patient (hour and minute documented by the laboratory technician in the protocol) and till the exact time when the test result occurred in the laboratory documentation system in the medical records (hour and minute).

### Statistics and ethics

The sample size was calculated assuming a reduction in the mean time used from collection to reporting from 60 minutes using tube transporting for conventional laboratory analysis to 30 minutes using the POCT and a standard deviation of 60 minutes in both groups, which required a sample size of at least 85 in each group. A p-value of ≤ 0.05 was chosen as significance level and a power of 90%.

The mean time used from collection to reporting was calculated in minutes for both groups (POCT and TTCL) and summary statistics were performed by means of unpaired Students’ t-tests.

To ensure that data would meet model requirements of normal distribution standardized normal probability plots were performed showing a p-distribution.

All analyses were performed using Stata, version 11 [7].

The study has been licensed by the Danish Data Protection Agency and needed no further local or national ethical approval according to Danish research legislation.

## Results

Blood samples from a total of 319 patients were included; 155 were randomized to POCT analysis (1 missing), and 164 samples were randomized to tube transporting for central laboratory analysis (5 missing), see Figure 1.

The mean time from collection to reporting was 24 minutes (SD=14.6, 95% CI=21.60-26.27 minutes) for the POCT group and 70 minutes (SD=32.9, 95% CI=64.54-74.85 minutes) for the TTCL group, i.e. a significant reduction in the mean time from collection to reporting using POCT of 46 minutes (p<.0001).

## Discussion

This study showed a significant 66% time reduction from 70 to 24 minutes from collecting to reporting time by using POCT compared to tube transporting blood samples for central laboratory analyses.

The results confirm our hypothesis that POCT delivers faster results than conventional TAT circles, even when optimized by high speed tube transporting system. To our knowledge, this is the first study comparing a newly developed high quality POCT device with a likewise newly developed transport system and central analysis. The result corroborates the results of other differently designed studies of POCT [2,8].

It might not be surprising that a TAT circle including a device with a shorter analysis time and an eliminated transport distance delivers faster answers compared to a traditional central laboratory device. However, our results estimate the magnitude of the time reduction to approximately 2/3.

Does this have any practical implication for the work flow in the ED considering the fact that the use of POCT is limited by several factors even when the quality is comparable with the central laboratory quality, especially the volume and variety of analysis and the costs? There are clinical situations where a significant time reduction to answers is much warranted. First, in situations where serious conditions are considered and the following diagnostic step could be chosen with a higher certainty when a laboratory test is available. In a patient with severe dyspnea a negative D-dimer rules out pulmonary embolism. In a patient with severe chest pain, the fast laboratory results might guide the physician in the direction of diagnosing procedures for aortic dissection (positive D-dimer) or myocardial infarction (positive troponins). Second, the situations where laboratory results enables the physician to discharge a patient to home without any further examinations, like ruling out deep venous thrombosis by negative D-dimer or abdominal discomfort where patients might be reassured after a skilled abdominal examination and a few normal laboratory results, such as CRP, HCG and hemoglobin.

In all these cases, a significant time reduction in waiting for laboratory result might save valuable minutes for the patient as well as the staff of the ED.

The present study is limited by certain factors. The time duration reflects the current working procedures which includes several steps in the POCT procedure as well as in the TTCL procedure. All though each step in both procedures was optimized as far as possible future improvements might still be possible. This is most likely to happen in the TTCL group, where more steps are involved, for instance automations in transferring the blood samples from the tube system to the analytic device and thus reduce the difference found. Furthermore, our study was only done on CRP. However since the analytic time is equal for other analysis, like D-dimer, HCG and troponins we do not consider this will change the results. The study was only performed when the project laboratory technicians were at work and did not include blood samples 24 hourly. There might be diurnal variations in TAT due to changes in the number of staff available, but due to randomization this would be equally distributed in both groups and not influence the analysis time difference found. We do not believe that the procedure time will be prolonged if POCT is used by non-laboratory staff, for instance nurses, due to the fact that the technology is easy to operate. A key question is whether the reduced time from collecting to reporting using POCT also results in shorter time to action as recent studies have suggested [1]. The fragmented test of time used from the blood sample was collected to the test result was reported using two different methods could be argued as another limitation of the study. Both methods are steps in complex and multifactorial organizational procedures and therefore the result might be difficult to implement in clinical practice without multiple analyses of each step in the process, as several other factors are involved and our study does not answer this question. Further research – and preferably randomised controlled trials with huge sample sizes – investigating the impact of a POCT reduced time from collection to reporting on time to decision and length of stay would be desirable, but so far, the documentation of this in the medical records is not sufficient to reliably answer this question.

## Conclusions

We found a significantly reduced mean time from collection to reporting using Point of Care Technology (POCT) in an emergency department compared to tube transported blood samples for central laboratory analyses. We recommend further research including the impact of time from collection to reporting on time to decision.

## Abbreviations

POCT: Point of care technology; ED: Emergency department; NPT: Near patient testing.

## Competing interests

There are no competing interests to be declared.

## Authors’ contribution

Both authors have contributed significantly to the work and approved the submitted manuscript. The manuscript has not been published or considered for publication elsewhere.

## Authors’ information

Birgitte Nørgaard is an MNSc, PhD, Post Doc in the Emergency Department, Kolding Hospital, a part of Lillebaelt Hospital. Her research is focused around post grade communication skills training, interprofessional team training, standardised timed patient pathways and health services research. Her recent publications are: Nørgaard B, Kofoed PE, Kyvik KO, Ammentorp J. Communication skills training for health care professionals improves the adult patient’s experience of quality of care. *Scandinavian Journal of Caring Sciences*.2012; doi: 10.1111/j.1471-6712.2012.00982.x Nørgaard B, Ammentorp J, Kyvik KO, Kofoed PE. Communication skills training increases self-efficacy of health care professionals. *The Journal of Continuing Education in the Health Professions 32*(2), *2012*. *DOI*:10.1002/chp.

Christian Backer Mogensen is an MD, PhD, Associate Professor in the Emergency Department, Kolding Hospital, a part of Lillebaelt Hospital. His research concerns clinical outcome parameters in acutely admitted patients and health services research. His recent publications are: Mogensen CB, Mortensen AMM, Staehr PB. Acute referral of patients from General Practitioners: Should the hospital doctor or a nurse receive the call? *Scandinavian Journal of Trauma*, *Resuscitation and Emergency Medicine 2011*, *19*:*47*, *doi*:10.1186/1757-7241-19-47;

Mogensen CB, Borch A, Brandslund I. Point of Care Technology or standard laboratory service in an Emergency Department: Is there a difference in time to action? A randomised trial. *Scandinavian Journal of Trauma*, *Resuscitation and Emergency Medicine* 2011, 19:49, doi:10.1186/1757-7241-19-49.
